# Methionine oxidation within the prion protein

**DOI:** 10.1080/19336896.2020.1796898

**Published:** 2020-08-02

**Authors:** John Bettinger, Sina Ghaemmaghami

**Affiliations:** Department of Biology, University of Rochester, Rochester, NY, USA

**Keywords:** Prions, oxidation, methionine, protein misfolding, protein aggregation

## Abstract

Prion diseases are characterized by the self-templated misfolding of the cellular prion protein (PrP^C^) into infectious aggregates (PrP^Sc^). The detailed molecular basis of the misfolding and aggregation of PrP^C^ remains incompletely understood. It is believed that the transient misfolding of PrP^C^ into partially structured intermediates precedes the formation of insoluble protein aggregates and is a critical component of the prion misfolding pathway. A number of environmental factors have been shown to induce the destabilization of PrP^C^ and promote its initial misfolding. Recently, oxidative stress and reactive oxygen species (ROS) have emerged as one possible mechanism by which the destabilization of PrP^C^ can be induced under physiological conditions. Methionine residues are uniquely vulnerable to oxidation by ROS and the formation of methionine sulfoxides leads to the misfolding and subsequent aggregation of PrP^C^. Here, we provide a review of the evidence for the oxidation of methionine residues in PrP^C^ and its potential role in the formation of pathogenic prion aggregates.

## Prion diseases and methionine oxidation

Prion diseases are a group of invariably fatal neurodegenerative diseases characterized by the self-templated misfolding of the cellular prion protein (PrP^C^) into insoluble protein aggregates (PrP^Sc^) in the central nervous system [1,2]. These diseases share etiologic features with other neurodegenerative diseases including Alzheimer’s and Parkinson’s diseases [3–5]. Prion diseases have been observed to occur naturally in several mammalian species including human and non-human primates, cervids, bovids, ovids, felines, minks and ungulates [6–14]. In addition to naturally occurring pathologies, prion diseases can be experimentally transmitted to a number of model organisms in laboratory settings [15–18]. Within the context of prion diseases, the process of protein aggregation can have varying modes of initiation and propagation. In sporadic Creutzfeldt Jacob Disease (sCJD), endogenously expressed PrP^C^ undergoes spontaneous misfolding and aggregation. In genetic prion diseases such as Fatal Familial Insomnia (FFI), heritable disease-associated mutations in PrP^C^ promote its aggregation. In acquired prion diseases such as variant CJD (vCJD), exposure to PrP^Sc^ from diet or other external routes initiates the PrP^C^ aggregation cascade [19–21]. sCJD is the most prevalent form of prion disease and occurs at a rate of roughly 1 to 1.5 cases per 1 million population per year [22]. Despite significant recent advances, there is currently no effective therapy against prion diseases and the mechanism of these diseases remains incompletely understood. Elucidating the cellular and genetic factors that promote the misfolding of PrP^C^ will greatly facilitate the development of novel therapeutics against these devastating diseases.

In acquired forms of prion disease, PrP conformers that seed the formation of PrP^Sc^ and initiate subsequent rounds of aggregation are introduced exogenously. In contrast, during the course of sporadic and inherited forms of the disease, the initial aggregation and formation of PrP^Sc^ seeds are thought to occur through destabilization and transient misfolding of endogenous PrP^C^ [22–28]. In some genetic prion diseases, pathogenic coding mutations have been shown to directly destabilize PrP^C^ and contribute to its aggregation [23]. However, most disease-associated mutations in PrP^C^ have not been directly linked to the destabilization of its folded structure, and factors that induce the initial destabilization and misfolding of PrP^C^ in sporadic and inherited forms of prion disease remain obscure [29,30]. Because of this, there is significant interest in characterizing environmental conditions that induce the destabilization of wildtype and mutated PrP^C^ and facilitate their aggregation. Among the conditions studied, oxidative stress and increases in reactive oxygen species (ROS) provide a compelling mechanism by which the initial misfolding of PrP^C^ can be induced under physiological conditions [31,32]. Indeed, increases in ROS production have been described as pathological features of prion diseases, as well as a number of other neurodegenerative disorders including Alzheimer’s disease, Huntington’s disease and Parkinson’s disease [31–35].

ROS are redox reactive metabolites that can oxidize amino acids in proteins and result in protein misfolding. The sulfur containing amino acid methionine has been demonstrated to be uniquely prone to oxidation by ROS, resulting in the formation of protein bound methionine sulfoxide residues [36]. Unmodified methionine is a non-polar hydrophobic amino acid whereas methionine sulfoxide is a polar hydrophilic amino acid. Among hydrophobic amino acids, methionine is the most polarizable [37]. The magnitude of dispersion forces that mediate the interaction between non-polar moieties is proportional to polarizability, suggesting that methionine and methionine oxidation can play an important role in establishing the dispersion forces that mediate protein folding and structure. The oxidation of methionine is therefore believed to impact protein structure by altering the hydrophobic forces that drive protein folding [37]. Indeed, in model peptides, it has been shown that the oxidation of methionine can act as a redox switch by converting alpha helical structures to beta-rich structures [38]. Peptides with alternating hydrophobic/hydrophilic side chains have a propensity to form beta sheet structures, whereas peptides with patterns of hydrophobic and hydrophilic side chains that match the alpha helical periodicity of 3.6 residues/turn have a propensity to form alpha helices [39–41]. Thus, the oxidation of methionine from a hydrophobic side chain to a hydrophilic side chain can potentially alter the patterns of hydrophobicity and hydrophilicity that govern secondary structure preferences. For example, Dado et al. were able to design an alpha helical peptide that transforms into a beta strand conformation under oxidizing conditions [38]. This conformational switch was driven by the formation of methionine sulfoxide residues that formed the hydrophilic face of a beta strand. Analogously, the transformation of PrP^C^ to PrP^Sc^ is associated with the conversion of alpha helical conformations to beta-rich aggregates and may therefore be similarly influenced by methionine oxidation [42]. Here, we review the evidence for the occurrence of methionine oxidation in PrP^C^ and its potential impact on prion formation.

## Methionines in PrP^C^

**Figure 1.**
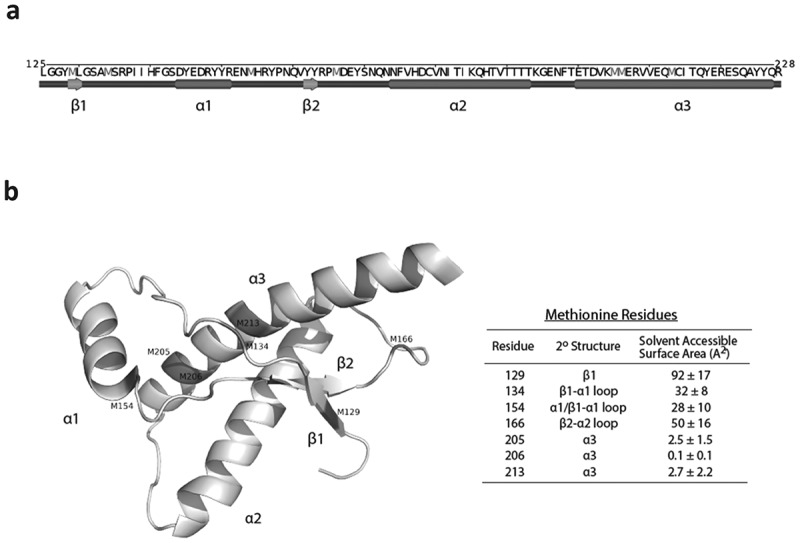
The location and structure of methionine residues in the structured C-terminal domain (125–228) of the human prion protein (huPrP). (a) A linear representation of the secondary structure content of huPrP generated by POLYVIEW [110]. The primary sequence of huPrP(125–228) is shown at the top and methionine residues are colored in magenta. Alpha helices are represented as red cylinders and beta strands are represented as green arrows. (b) The tertiary structure of huPrP(125–228) (PDB:1QLX) [44]. Methionine residues are labelled by their residue number in the human prion protein. Solvent exposed methionines are shown in blue and buried/partially buried methionines are shown in red. Solvent accessible surface areas (SASAs) were calculated in pyMol with sampling density set to 3. SASA values are represented as a mean and standard deviation derived from six unique solution NMR structures (PDB: 1QLX,1QLZ,1QM0,1QM1,1QM2,1QM3) [44].

The cellular prion protein (PrP^C^) is a predominately alpha helical protein with an unstructured N-terminal domain and a structured C-terminal domain (Figure 1). The C-terminal domain is sufficient to form infectious PrP^Sc^ aggregates and consists of three alpha helices (amino acids 144–154, 175–193 and 200–219) and a small antiparallel β-sheet (amino acids 128–131 and 161–164) [43,44]. A disulfide bond (C179–C214) links helices 2 and 3 [43,44]. There are two sites of glycosylation in PrP^C^ that are modified with diverse N-linked glycans [45,46]. The predominant form of PrP^C^ also contains a glycosylphosphatidylinositol (GPI) moiety that anchors it to the cell membrane at its C-terminus.

Human PrP contains 12 methionines in total, including the initiator methionine. The methionine content of PrP is unusually high in comparison to the rest of the proteome where methionine is the second rarest amino acid residue with a prevalence of only 1.8% in vertebrate proteins [47]. In PrP, seven methionine residues are found within the structured C-terminal domain that forms the core of protease-resistant PrP^Sc^ aggregates (Figure 1b). Within these methionines, M129, M134, M154, and M166 are surface-exposed and are thus more vulnerable to oxidation by ROS whereas M205, M206 and M213 are partially or completely buried [44]. The residue M129 is polymorphic in human prion sequences, existing as predominately M129 or V129. Homozygosity and heterozygosity at position 129 has been demonstrated to impact the progression and pathological presentation of prion diseases [48].

## Replacement of methionines with polar residues disrupts the structure and stability of PrP^C^

Evidence for methionine oxidation in PrP has been historically well documented [49]. The detection of methionine oxidation in PrP, and the growing interest in the intersection between oxidative stress and neurodegenerative diseases, has led many researchers to investigate the potential role of methionine oxidation in the pathogenesis of prion diseases. To facilitate these studies, a number of laboratories have developed methodologies for simulating or inducing methionine oxidation in PrP^C^ and studying its impact on protein structure and stability.

In their study, Elmallah et al. simulated methionine oxidation of PrP^C^ by replacing methionines in PrP with serine residues [50]. Although serine has a similar structure to methionine, the polarity of its side chain is closer to that of methionine sulfoxide. By replacing methionines with serines and measuring the resulting shifts in folding stabilities, Elmallah et al. were able to quantify the structural impact of additional polarity within methionine encoded positions of PrP^C^. It was found that serine replacement of all surface methionine residues induced the formation of stable folding intermediates that have molten globule-like character. Individual serine replacement of the polymorphic site 129 did not significantly impact the structural stability of the prion protein; suggesting that additional polarity at the polymorphic site 129 does not significantly contribute to the structural destabilization of PrP^C^. The observed molten globule-like structures formed, following total replacement of all surface methionines, were found to be similar to that of previously described misfolding intermediates of PrP^C^ [51–55]. However, it was not clear if the folding intermediate of PrP^C^ observed following serine replacement of surface methionines reflects a structure that is on the aggregation pathway of PrP. The authors of this study suggest that the structure of the folding intermediate formed following serine replacement of surface exposed methionines causes a structural rearrangement of the buried methionine residues 205 and 206, rendering them more susceptible to oxidation by ROS. Further oxidation of PrP^C^ intermediates formed following serine replacement of all surface exposed methionines induced the formation of a new stable oligomeric state. The structure of this intermediate is similar in size and stability to intermediates previously described as being on the aggregation pathway of PrP^C^, suggesting a role for the oxidation of both surface exposed and non-surface exposed methionines in the aggregation of PrP^C^ [51–55].

Other studies on the effect of methionine to serine substitutions on the structure and stability of PrP^C^ confirm that while serine replacement of both surface-exposed (M134, M154) and buried methionines (M206, M213) alter the structure and thermodynamic stability of PrP^C^, serine replacement of buried methionines 206 and 213 have a much larger impact [56]. In addition, it has been observed that serine replacement of M206 and/or M213 results in a higher aggregation propensity compared to wildtype PrP, based on thioflavin T (ThT) fluorescence kinetics during a prion conversion assay [56].

In addition, molecular dynamics (MD) studies have shown that substitution of M205 with polar amino acids serine and arginine causes structural distortions in PrP^C^ [57]. These MD simulations suggest that the hydrophobic character of M205 is required to stabilize the interaction between alpha helices 1 and 3. Position 205 is a well-conserved amino acid in PrP and is invariably occupied by a hydrophobic amino acid. Large-scale mutational studies have confirmed the structural importance of a hydrophobic residue at position 205 [58]. Taken together, these results suggest a model in which the oxidation of surface exposed methionines on PrP^C^ induce the early stages of prion misfolding, and subsequent oxidation of buried or partially buried methionine residues 205, 206 and 213 plays a role in further destabilization of PrP^C^ structure and formation of aggregates.

Similar studies to the ones discussed above have confirmed the structural intolerance of PrP^C^ towards polar substitutions within methionine encoded residues. Wolschner et al. used a strategy of replacing methionine residues in PrP with analogues norleucine (Nle) and methoxine (Mox) [51]. Nle is non-polar and non-oxidizable, whereas Mox is polar and non-oxidizable. Thus, Nle and Mox can be described as non-oxidizable analogs of methionine and methionine sulfoxide, respectively. It was found that Nle-PrP formed a structurally stable monomeric form that was rich in alpha helical content and lacked the *in vitro* aggregation properties of wildtype PrP^C^. Conversely, Mox-PrP formed an unstable and beta-rich structure that was more aggregation-prone than wildtype PrP^C^. Both unoxidizable variants of PrP^C^ showed aggregation properties that had a weaker response to oxidant treatment compared to that of the parent wildtype PrP^C^.

## Oxidation of methionines disrupts the structure and stability of PrP^C^

A number of studies have demonstrated a direct correlation between methionine oxidation and PrP^C^ misfolding and aggregation. In their study, Wolschner et al. observed a correlation between the extent of methionine oxidation, as monitored by electrospray mass spectrometry, and aggregation of PrP^C^ induced by periodate [51]. Lower concentrations of periodate were sufficient to oxidize surface methionines and caused moderate aggregation of PrP^C^, whereas higher oxidant concentrations were able to further oxidize the buried methionines and caused severe aggregation of PrP^C^.

In another study, it was confirmed that oxidation of PrP^C^ by mild hydrogen peroxide treatment causes a decrease in the thermodynamic stability of PrP^C^ by as much as 9 kJ/mol as measured by urea denaturation experiments [53]. Interestingly, it was observed by size exclusion chromatography that hydrogen peroxide treated PrP^C^ retains a largely monomeric form. A combination of mass spectrometry and circular dichroism experiments confirmed that treatment of PrP^C^ with hydrogen peroxide results in the rapid oxidation of methionine residues, with the extent of oxidation correlating with solvent exposure, and that hydrogen peroxide treated PrP^C^ retains a largely alpha helical conformation with a slight increase in beta strand content [59]. However, it was observed by 2D NMR chemical shift perturbations (CSPs) that the structures of unoxidized and hydrogen peroxide treated PrP^C^ are distinct from one another. CSP analysis also demonstrated that methionine residues were the most extensively modified amino acids following hydrogen peroxide treatment as most non-methionine residues showed little CSP following oxidation. The exceptions were non-methionine residues that were clustered within the vicinity of modified methionine residues and are part of the hydrophobic core of PrP^C^ [53]. These residues include Val209, Val160 and Tyr156 that are tightly packed against buried methionines 206 and 213 in helix 3 of PrP^C^.

MD simulations confirm that the *in silico* conversion of methionine to methionine sulfoxide of either or both M206 and M213 causes structural perturbations to the native structure of PrP^C^ [60]. These perturbations include increased flexibility within the region encompassing helix 3 and portions of helix 2 [60]. Interestingly, this region of PrP^C^ has been described as one of the structural ‘hotspots’ of prion conversion [61,62]. In addition, the loop connecting helix 2 and helix 3 is identified as the binding site of the prion chaperone compound GN8 [63]. These results suggest that oxidation of core methionine residues may promote structural perturbations in the regions of PrP that are critical for aggregation and prion conversion.

When hydrogen peroxide-treated PrP^C^ is further oxidized by exposure to ROS produced by copper catalysis, more profound conformational alterations are observed. Whereas the structure of PrP^C^ treated with hydrogen peroxide alone retained much of its native structure, PrP^C^ treated with both hydrogen peroxide and copper lost approximately one third of its amide signal in 2D NMR analysis [53]. The majority of amide signal loss was attributed to residues within helix 2 and helix 3, strongly agreeing with MD simulation results suggesting increased flexibility within this region following oxidation of M206/213 [60]. Circular Dichroism (CD) and ANS staining confirm that oxidation of PrP^C^ with both hydrogen peroxide and copper results in the formation of a molten globule-like protein with alpha helical content [53]. The 2D NMR spectra of PrP^C^ following oxidation by both copper and hydrogen peroxide is highly similar to that of an alternative structure of PrP^C^ formed under mildly acidic conditions (pH 4.1) [53,55,64].

Oxidation of PrP^C^ by a combination of hydrogen peroxide and copper treatment under harsher conditions produced a highly unstable intermediate of PrP^C^ with high beta sheet content that others have shown to have a tendency to form oligomers [52]. This behaviour is reminiscent of the behaviour of PrP^C^ in harshly acidic environments (pH 3.5) [53,55,64]. These results suggest a general mechanism of prion misfolding under oxidative and acidic conditions. In addition, experimental evidence supports the observation that copper treated PrP^C^ aggregates into beta sheet-rich oligomers that are similar in size and shape to oligomeric species previously described as being highly infectious. These results suggest a direct link between methionine oxidation and pathogenic misfolding of PrP [52,65,66].

Interestingly, it has also been observed that methionine oxidation may directly impact the aggregation pathway of PrP^C^. In studies of amyloidgenic peptides, it was found that the amyloidogenic properties of the PrP fragment peptide (106–126) were significantly decreased by oxidation [67]. In addition, Breydo et. al have shown that methionine oxidation of full-length recombinant mouse and hamster PrP inhibited amyloid fiber growth [68]. It was observed that the inhibitory effects of methionine oxidation on amyloid fiber growth were stronger for hamster PrP than they were for mouse PrP. The authors of this study attribute the species-specific difference to three additional surface exposed methionine residues in the hamster prion protein. In the context of prion diseases, the amyloid fibers themselves may not be the cytotoxic species. Rather, it has been suggested that small oligomeric intermediates formed on pathway to amyloid fibers are the true cytotoxic species in prion diseases [69]. Breydo et. al suggest that methionine oxidation may interfere with amyloid fiber formation and as a consequence increase the population of cytotoxic oligomers [68].

## Methionine oxidation of disease-associated mutants of PrP

The studies discussed above provided important insights into the impact of methionine oxidation on the structure, stability and aggregation of wildtype prion protein and suggest a role for methionine oxidation in the progression of sporadic prion diseases. There is also increasing evidence that methionine oxidation may play a role in the pathogenesis of genetic prion diseases that are associated with specific pathogenic mutations in the human prion protein. There are more than 35 mutations in the human prion protein sequence that have been linked with genetic prion diseases [70]. A complete survey of methionine oxidation in all disease-associated mutant prion sequences is not yet available. However, studies on the E200K and D178N variants, which are the first and third most common disease-causing prion mutations, suggest a possible role for methionine oxidation in the progression of genetic prion diseases [71].

The E200K mutation of huPrP that is associated with genetic Creutzfeldt-Jakob disease (gCJD), places a negatively charged amino acid in place of a positively charged amino acid at the beginning of helix 3 (Figure 1a). The E200K mutation is the most common mutation associated with genetic prion diseases [72]. However, it has been shown that the E200K mutation in itself does not significantly alter the structure or stability of PrP^C^ [29,30,73]. There has therefore been great interest in understanding the mechanisms by which the E200K mutation contributes to the etiology of genetic prion diseases. It has been suggested that the E200K variant of huPrP may be more prone to methionine oxidation *in vivo* than wildtype huPrP [74]. Canello et al. show that while an antibody (pAB RVC) specific to helix 3 buried methionines (M205/206/213) was able to readily detect huPrP in brain homogenates of healthy individuals and patients of sporadic prion disease, PrP in brain homogenates of individuals heterozygous for E200K was poorly detected [74]. The peptide used to generate pAB RVC did not include position 200, precluding it from the epitope regardless of the residue present in that position. Furthermore, it was demonstrated that methionine oxidation inhibits PrP recognition by pAB RVC and the specific reduction of methionines by N-methylmercaptoacetamide (MMA) treatment restores recognition. The authors of this study suggest that the poor detection of E200K huPrP in brain homogenates is a result of methionine oxidation. In support of this, recombinant E200K huPrP, but not recombinant wildtype huPrP, was recognized by an antibody (pAb DZS18) raised against a methionine sulfoxide rich maize protein that has been previously shown to recognize methionine oxidation in proteins [74,75]. These results suggest that helix 3 methionines in E200K huPrP may be more prone to endogenous oxidation than wildtype huPrP.

It has also been demonstrated that *in vitro* methionine oxidation of E200K huPrP occurs in a site-specific sequential manner and results in the conformational conversion of PrP^C^ into proteinase-K resistant aggregated conformations. Wang et al. demonstrated that incubating E200K huPrP with 50 mM hydrogen peroxide for 2 hours was sufficient to cause near complete oxidation of all surface methionines (M109/112/129/134/154/166), but the oxidation of buried methionines (M205/206/213) required a longer incubation period of 12 hours [76]. Using CD spectroscopy, it was shown that incubation of E200K huPrP with 50 mM hydrogen peroxide for 2 hours had little impact on its secondary structure content, as the oxidized protein retained its predominantly alpha helical structure. However, thermal and urea denaturation experiments indicated that the oxidation of E200K huPrP for 2 hours results in a significant decrease in its stability. These results suggest that surface methionines in E200K huPrP are the first to become oxidized upon exposure to hydrogen peroxide, and although this initial oxidation results in minimal structural perturbations, it contributes to the destabilization of PrP^C^. Further oxidation of E200K huPrP with 50 mM hydrogen peroxide for an additional 10 hours was sufficient to cause oxidation of buried methionines and a major structural rearrangement of E200K huPrP, characterized by an increase in beta sheet content. In addition, oxidation of E200K huPrP for 12 hours resulted in a significant drop in protein stability compared to both unoxidized E200K huPrP and E200K huPrP that had been oxidized for only 2 hours. Mutational studies suggested that the oxidation of M213 was required for the structural conversion of E200K huPrP to a predominately beta-sheet structure. These results suggest that the oxidation of surface methionines may induce the early stages of PrP misfolding and that further oxidation of buried methionines, in particular M213, is sufficient to cause a total structural rearrangement as well as a significant decrease in the stability of mutated PrP. Indeed, it was shown that E200K huPrP that has been oxidized for 12 hours had increased proteinase K resistance compared to unoxidized E200K huPrP. In contrast, E200K huPrP that has been oxidized for only 2 hours showed no significant difference in proteinase K resistance compared to unoxidized huPrP. It is important to note that proteinase K-resistant and methionine oxidized forms of E200K huPrP have also been reported in the brains of transgenic mouse models of prion disease [77].

The D178N variant of PrP, associated with fatal familial insomnia (FFI) and genetic Creutzfeldt-Jakob disease (gCJD), has also been suggested to be more prone to methionine oxidation than wildtype PrP [71,78]. It was shown by mass spectrometry that after short periods of incubation with 50 mM hydrogen peroxide, all surface methionines in both wildtype PrP and the D178N variant become fully oxidized [78]. The complete oxidation of surface methionines in the D178N variant occurred within 1 hour, whereas it took approximately 2 hours to achieve the same in wildtype PrP. Limited oxidation of buried methionines 205/206 and the partially exposed methionine 213 was observed after 8 hours of incubation with hydrogen peroxide in the D178N variant and after 20 hours in wildtype PrP. Together, these results suggest that both surface and buried methionines are more prone to oxidation in the D178N variant. Partial oxidation of buried methionines was found to be sufficient to cause a structural conversion in D178N PrP towards a predominately beta-sheet structure, as judged by CD analysis. In contrast, wildtype PrP showed minimal structural alteration after the same oxidation period. Furthermore, ANS fluorescence intensities demonstrated that the oxidized form of D178N PrP has more exposed hydrophobic surface area than that of oxidized wildtype PrP, suggesting that it may be more prone to aggregation. Indeed, it was shown that the D178N variant of PrP forms oligomers and aggregates following hydrogen peroxide treatment more rapidly than oxidized wildtype PrP. In agreement with this result, oxidized D178N PrP was shown to be more resistant to proteinase K digestion than oxidized wildtype PrP. Additionally, N2a cells transfected with the D178N variant under conditions of oxidative stress had a higher content of prion aggregates compared to N2a cells transfected with wildtype PrP under the same conditions. Apoptosis was also found to be elevated in N2a cells transfected with D178N under conditions of oxidative stress compared to N2a cells transfected with wildtype PrP under the same conditions. Taken together, these results suggest that the D178N variant of PrP is more vulnerable to oxidation-induced aggregation than wildtype PrP.

Methionine oxidation of the polymorphic variant D178N/M129V, associated with distinct pathological subtypes of prion disease, did not result in significant differences in aggregation when compared to the D178N variant, suggesting that polymorphisms at residue 129 do not significantly contribute to the oxidation induced aggregation of PrP^C^. Thus, methionine oxidation under conditions of oxidative stress may contribute to the progression of some genetic prion diseases regardless of the amino acid at position 129.

## Methionine oxidation as a clinical feature of prion disease

The data described above suggest that the artificial oxidation of methionine residues can contribute to the misfolding and aggregation of PrP. However, whether methionine oxidation is a standard clinical feature of prion diseases remains somewhat controversial. Evidence for methionine sulfoxides in PrP^Sc^ isolated from diseased tissues was first described nearly 30 years ago [79]. However, because of the potential for artifactual *in vitro* oxidation of methionines during stages of sample preparation and analysis, it was unclear whether this observation was indicative of high levels of methionine sulfoxides within PrP^Sc^ *in vivo* [80–83]. Indeed, the issue of potentially artifactual *in vitro* oxidation of methionine residues is at the core of the controversy surrounding measurements of methionine oxidation in PrP^Sc^. As described below, some studies have found strong enrichment for methionine oxidation in prion infected samples while others report no significant differences in the extent of PrP oxidation between normal and prion infected samples [36,74,84–86].

Canello et. al describe a method for quantifying methionine oxidation in PrP by using antibodies that are specific towards unoxidized methionine residues in PrP [84]. The antibody mAB IPC2, raised against mouse PrP, has broad specificity and is able to recognize recombinant PrP from a variety of species. Mutational studies suggested that the substitution M213S hindered the recognition of PrP by IPC2. *In vitro* studies additionally demonstrated that pretreating PrP with hydrogen peroxide hindered recognition of PrP by IPC2, and the specific reduction of methionines by MMA treatment was able to restore this recognition. The authors of this study conclude that the oxidation of methionines, in particular M213, can be inferred from the binding properties of IPC2. IPC2 was subsequently used to study the extent of methionine oxidation in PrP^C^ and PK-resistant PrP^Sc^ prepared from brain homogenates. It was found that in comparison to PrP^C^, methionine oxidation was enriched in PrP^Sc^ derived from brain homogenates of mice infected with the RML prion strain, Syrian hamsters infected with the sc237 prion strain, and human samples from sporadic and E200K associated CJD patients. The strongest enrichment was found in human prion samples and almost no evidence for methionine oxidation was detected in the corresponding PrP^C^ fractions. Subsequent studies confirmed that IPC2 was unable to recognize PrP^Sc^ from human prion samples, but was able to recognize a fraction of PK-resistant PrP^Sc^ from mouse prion samples [74]. Recognition of PK-resistant PrP^Sc^ by IPC2, in both mouse and human samples, was restored by MMA treatment. The authors concluded that some strains of PK-resistant PrP^Sc^ are enriched for methionine sulfoxides and suggested that methionine oxidation may represent a covalent signature of prion diseases.

Conflicting results have been obtained by others using a different strategy for the quantification of methionine oxidation. Silva et al. developed a quantitative mass spectrometry-based multiple reaction monitoring (MRM) assay for methionine oxidation of M213 in a Syrian hamster model of prion disease [85]. Oxidation of M213 was monitored over the time course of intercranial challenge with a hamster adapted strain of scrapie (263K). It was found that methionine sulfoxide content of M213 remained low throughout the course of intercranial challenge and decreased overtime. Additionally, it was found that the oxidation levels of M213 in PrP^Sc^ fractions of brain homogenates remained low and were comparable to methionine oxidation levels of PrP^C^ fractions. These results were confirmed for three strains of hamster adapted scrapie (263K, 139H and drowsy). Studies employing a similar methodology suggested that polymorphisms in the cervid prion sequence significantly impact the amount of M216 oxidation (analogous to M213 in the human sequence) observed in PrP purified from naturally infected cervids, with the identity of the amino acid in position 218 strongly correlating with observable M216 oxidation [86]. However, it was found that oxidation of M216 was not significantly associated with PrP^Sc^. The authors of this study suggest that oxidation of M213 does not represent a covalent signature of prion diseases and methionine oxidized PrP is not specifically enriched in prion aggregates.

It is possible that the conflicting results described above reflect the heterogeneity of prion strains or tissue samples investigated in the different studies. Alternatively, these discrepancies may be an artifact of technical limitations that have historically hampered accurate quantification of methionine oxidation from *in vivo* derived samples. For example, Silva et al. suggest that previous measurements of PrP^Sc^ methionine oxidation may have been significantly biased by the accumulation of methionine oxidation during gel electrophoresis and sample handling [81,85]. To minimize this problem, these authors prepared a standard curve and validated the quantitative accuracy of their assay using artificial variants of the target peptide with known levels of methionine oxidation, making their measurements less vulnerable to experimental artifacts [85]. However, it should be noted that this approach does not fully mitigate the issue of artifactual oxidation as mass spectrometric analysis in itself can result in spurious oxidation of methionines [82,83]. Thus, artificially prepared standards of the target peptide, as well as the target peptide itself, may both be vulnerable to the accumulation of artifactual methionine oxidation during mass spectrometric analyses and this may result in ratio suppression when comparing two samples such as PrP^C^ and PrP^Sc^ fractions.

**Figure 2.**
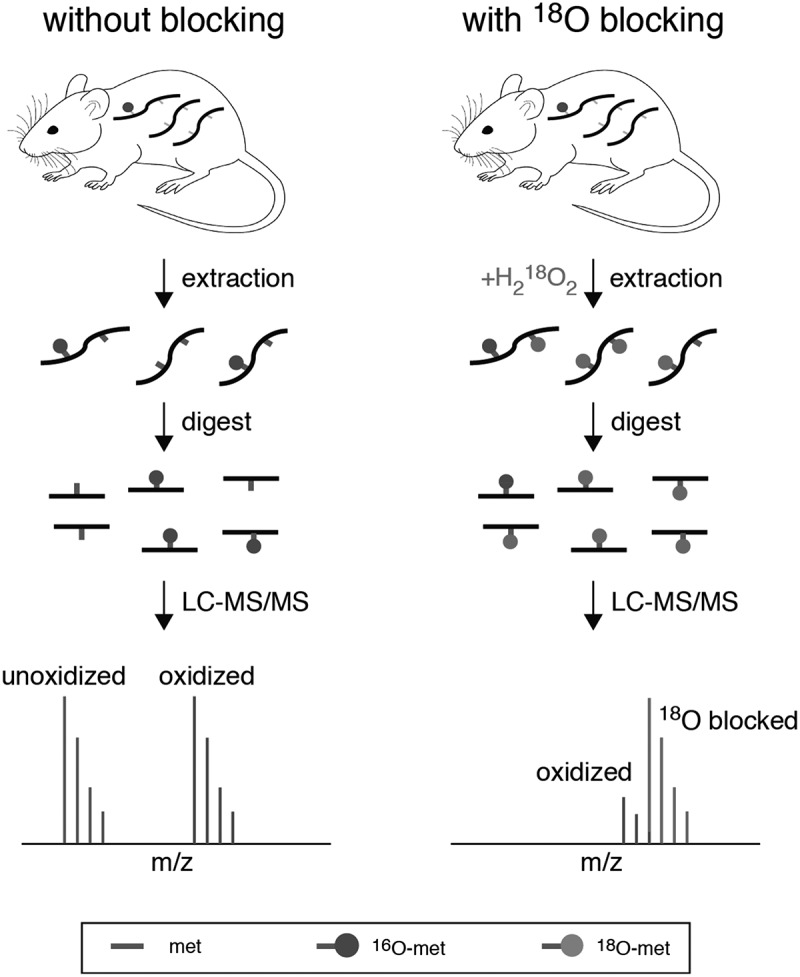
A schematic overview of a novel method for the accurate quantification of methionine oxidation [36]. As shown in the left panel, methionine oxidation can typically occur *in vitro* during sample preparation, and the measured levels of oxidation may not be reflective of levels of methionine oxidation *in vivo*. This problem can be solved by blocking unoxidized methionines with heavy-labelled ^18^O (right panel). At the time of extraction, unoxidized methionine residues are converted to methionine sulfoxide residues with a heavy oxygen atom label (red). The heavy oxygen label serves as a blocking agent and prevents the *in vitro* accumulation of methionine sulfoxides, labelled with a naturally occurring light oxygen atom (green). The differences in mass between heavy and light oxygen can be used to distinguish *in vivo* methionine oxidation from *in vitro* blocking by mass spectrometry. The resulting isotope clusters can then be deconvoluted and quantified by custom algorithms, resulting in a measurement of *in vivo* fractional oxidation.

It should be noted that newer methodologies have since been developed that circumvent artifactual oxidation by ‘blocking’ unoxidized methionines using isotopically labelled oxidizing agents prior to mass spectrometric analysis (Figure 2) [36,87,88]. This approach allows for accurate quantitation of *in vivo* methionine oxidation from tissue or cell extracts. The utilization of this technique may help clarify the significance of methionine oxidation as a clinical feature of prion diseases.

## The role of PrP in oxidative stress tolerance

**Figure 3.**
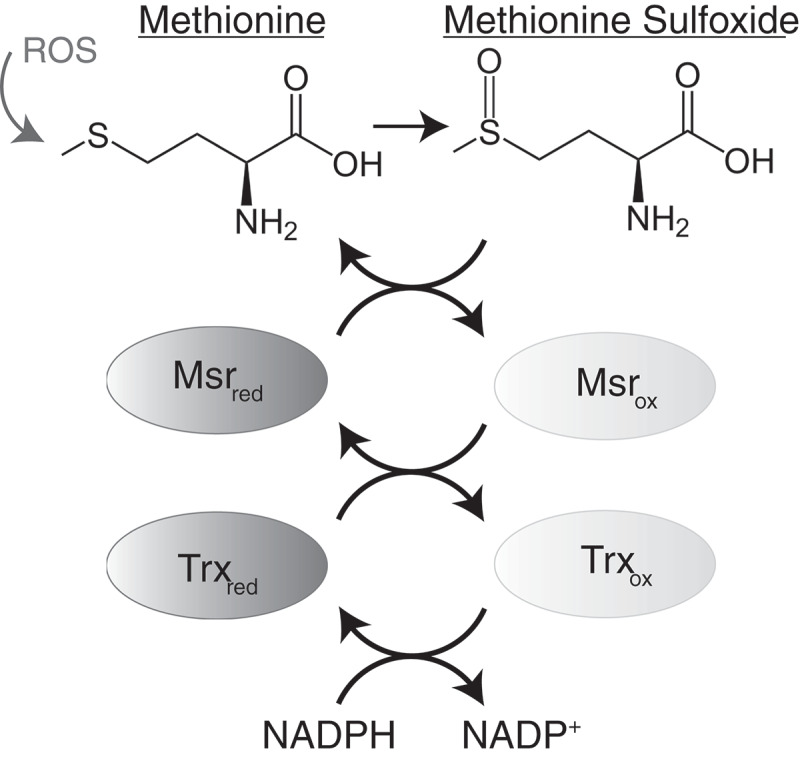
The methionine redox cycle. Methionine can be oxidized by reactive oxygen species (ROS) resulting in the formation of methionine sulfoxide. Methionine sulfoxide can be repaired by the action of methionine sulfoxide reductase (Msr) enzymes, resulting in a disulfide cysteine intermediate of Msr. Catalytic activity of Msr is restored by the action of thioredoxin (Trx) and thioredoxin reductases, using dihydronicotinamide-adenine dinucleotide phosphate (NADPH) as a cofactor, producing one molecule of nicotinamide adenine dinucleotide phosphate (NADP^+^).

Although the studies described above suggest a direct link between methionine oxidation and prion misfolding and aggregation, it has also been proposed that the oxidation of methionine residues in PrP may play a more indirect role in the pathology of prion diseases. Unlike most forms of protein oxidative damage, methionine oxidation can be reversed by the action of a system of specialized enzymes known as the methionine sulfoxide reductases (Msr) (Figure 3) [89–91]. Methionine sulfoxide reductases are a family of enzymes that reduce methionine sulfoxide residues back to methionines in a stereospecific manner [92]. Msrs can be divided into two subfamilies based on the stereospecificity of their catalytic activity: MsrAs that reduce the S-enantiomer of methionine sulfoxide and MsrBs that reduce the R-enantiomer of methionine sulfoxide. The Msr pathway consists of the concerted activities of Msrs, thioredoxin and thioredoxin reductases [93]. Msrs act as an electron donor to reduce protein-bound methionine sulfoxide residues. The products of this reaction are repaired (reduced) methionine residues and disulfide cysteine intermediates of Msrs. Catalytic activities of oxidized Msrs are subsequently restored by the reduction of disulfide cysteine intermediates by thioredoxin and thioredoxin reductases, with NADPH acting as the terminal electron donor to produce one molecule of NAD^+^ per catalytic cycle (Figure 3) [93]. Thus, it has been proposed that the oxidation of endogenous protein-bound methionine residues, in concert with the action of Msrs, constitutes a complete redox cycle that depletes intracellular ROS while maintaining the chemical integrity of methionine residues in proteins [94,95].

The high content of surface exposed methionines within the structure of PrP has been suggested to play a role in the endogenous antioxidant defense of neurons that express PrP. Similar to most neurodegenerative disorders, prion diseases are associated with a significant increase in ROS and oxidative stress [96,97]. Several studies have demonstrated that cells deficient in the expression of PrP are more sensitive to oxidative stress [98]. Early reports demonstrated that PrP^C^, produced recombinantly or purified form mouse brains, binds copper ions *in vitro* and has superoxide dismutase-like activity that inhibits the accumulation of ROS [99,100]. Subsequent reports demonstrated that PrP deficient mice have a neuronal phenotype that is more sensitive to oxidative stress [101]. These reports demonstrated that neurons cultured from PrP-deficient mice have altered expression of transcription factors and proteins involved in oxidative stress tolerance. These observations suggested that mice deficient in PrP have altered cellular metabolism that may compensate for the loss of anti-oxidant function of PrP. Similar observations have been made in PrP-deficient cultured cells [102]. These cells display decreased oxidative stress tolerance that can be rescued by treatment with the antioxidant molecule vitamin E. Together, these studies indicate that PrP has endogenous anti-oxidant activity in cultured cells and mouse model systems.

More recent reports have suggested that the antioxidant mechanism of copper-bound PrP^C^ involves the metal-catalyzed modification of amino acids near the copper binding sites. Residues within the unstructured N-terminal domain, M109/112 and His96/His111, were reported as the residues most vulnerable to metal-catalyzed modifications [103]. The authors of this study suggested that the oxidation of methionine and histidine residues in PrP^C^ act as sacrificial oxidants to limit the diffusion and reactivity of copper generated ROS. As described above, unlike histidines, the sacrificial oxidation of methionines can be reversed by Msrs, restoring the antioxidant activity of PrP^C^.

Interestingly, neurons cultured from PrP-deficient mice have SOD-1 activities that are unresponsive to oxidative stress, suggesting a role for PrP in the adaption of SOD-1 activity to oxidative stress [102]. Due to different subcellular localizations of SOD-1 and PrP^C^, the authors of this study suggested that PrP^C^ may play an indirect role in the regulation of SOD-1 activity. Later reports demonstrated that the regulation of SOD activity by PrP^C^ is dependent on the interaction between the octapeptide repeat (OR) region and the N-terminal half of the hydrophobic region (HR) of PrP and stress inducible protein 1 (STI1) [104,105]. The authors of these studies suggest that the mechanism of neuronal cell death during the course of prion disease may, at least in part, be due to a loss of oxidative stress tolerance. Thus, PrP^C^ may promote oxidative stress tolerance of neurons through two independent mechanisms. First, PrP^C^ may directly enhance oxidative stress tolerance by utilizing methionine residues as sacrificial oxidants that can be recycled by the Msr pathway. Second, PrP^C^ may play an indirect role in oxidative stress tolerance by regulating SOD activity. Future studies aimed at quantifying the relative contributions of these two functions may shed light on the role of oxidative stress tolerance in the progression of prion diseases.

It should be noted that a study of transgenic mice lacking the MsrA gene suggested that MsrA does not play a role in prion pathogenesis [106]. This study observed no difference between incubation periods of wildtype and MsrA knockout mice following inoculation with mouse adapted prion strains RML, Me7, and 301V. Additionally, further impairment of the Msr system by selenium deprivation, which lowers the levels of MsrB, showed no significant difference between the incubation times of MsrA knockout mice and wildtype mice [106,107]. These results suggest that the Msr system may not play a significant role in the progression of exogenously transmitted prion diseases. However, the potential role of the Msr system in the progression of sporadic or inherited prion disease remains unexplored. Analogous connections between Msr activity and disease pathology have been observed for a number of other neurodegenerative disorders. For example, MsrA knockout mice have been previously described as having an elevated vulnerability to sporadic brain pathologies, including increased deposition of Aβ peptide and hippocampal neurodegeneration [108].

## Concluding remarks

**Figure 4.**
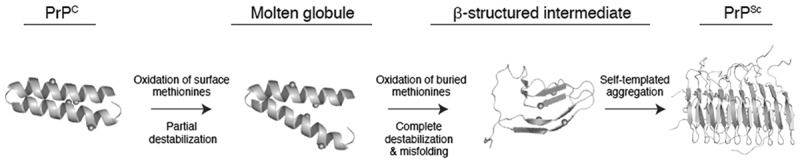
A proposed model for the role of methionine oxidation in prion protein misfolding and aggregation. Unoxidized methionines are represented as green circles and oxidized methionines are represented as red circles. Surface methionines in PrP can become oxidized resulting in the formation of a molten globule state and increasing the solvent exposure of buried methionines. In the molten globule state, buried methionines can become oxidized, resulting in the total destabilization and conformational rearrangement of the prion protein. It is unknown whether or not the β-structured intermediate induced by methionine oxidation is on pathway to the formation of PrP^Sc^. Schematic representations of PrP^C^ and the molten globule state were derived from PDB:1QLX [44]. Schematic representations of PrP^Sc^ were derived from PDB:2RNM [111].

Recent advances have provided a detailed understanding of the effects of methionine oxidation on the structure and stability of PrP^C^. A number of *in vitro* studies have demonstrated that methionines in PrP^C^ are vulnerable to oxidation. Methionine oxidation alters the structure of PrP^C^ and generally decreases its stability. The consensus mechanism that has emerged from these studies is that the oxidative misfolding of PrP^C^ likely begins by the oxidation of surface methionines and proceeds through subsequent oxidation of buried or partially buried methionines. The oxidation of buried methionines is required for the complete destabilization of PrP^C^ and conversion to beta-rich aggregated structures (Figure 4). However, bridging *in vitro* and *in vivo* studies of methionine oxidation in PrP has been somewhat more tenuous. Whereas some *in vivo* studies suggest that methionine oxidation is significantly associated with PrP^Sc^, other studies have found no such association. Furthermore, the *in vivo* evidence reviewed here is insufficient to assign methionine oxidation a causative role in the formation of prion aggregates. The levels of *in vivo* methionine oxidation observed in the prion protein during the course of prion infection may instead be a relatively benign side effect of increased oxidative stress. Additionally, the potential for methionine oxidation to impact the formation of distinct prion strains remains a largely unexplored topic. Previous reports have demonstrated that methionine oxidation impacts the stability, dynamics and structure of mature amyloid fibrils and protofibril aggregates formed from Aβ peptide (1–42), suggesting that methionine oxidation may play a similar role in dictating the formation of prion strains [109]. Future analyses of *in vivo* methionine sulfoxide levels within a range of prion strains and host species using more accurate analytical methodologies may help clarify the role of methionine oxidation in the pathology of prion diseases. Additionally, a greater understanding of the role of the Msr system in the progression of sporadic and inherited prion diseases will shed light on the role of PrP^C^ as an endogenous antioxidant. Together, these studies could significantly advance our understanding of the pathogenic mechanism of prion diseases and facilitate the development of new therapeutic strategies.
